# Whey Proteins-Fortified Milk with Adjusted Casein to Whey Proteins Ratio Improved Muscle Strength and Endurance Exercise Capacity without Lean Mass Accretion in Rats

**DOI:** 10.3390/foods11040574

**Published:** 2022-02-16

**Authors:** Eun Woo Jeong, Gyu Ri Park, Jiyun Kim, Youjin Baek, Gwang-woong Go, Hyeon Gyu Lee

**Affiliations:** 1 Department of Food and Nutrition, Hanyang University, Seoul 04763, Korea; bravoadria@hanyang.ac.kr (E.W.J.); opps112@hanyang.ac.kr (G.R.P.); kjy0601@hanyang.ac.kr (J.K.); jyyj161126@hanyang.ac.kr (Y.B.); 2 Korean Living Science Research Center, Hanyang University, Seoul 04763, Korea

**Keywords:** milk protein, endurance exercise capacity, muscle strength, whey proteins-fortified milk, casein to whey proteins ratio

## Abstract

This study investigated the effects of the casein to whey proteins (CW) ratio in milk on body composition, muscle strength, and endurance exercise capacity in rats. Thirty rats were assigned into five groups, and each treatment was administered for eight weeks: (1) control (isocaloric lactose supplementation), (2) CW8:2 (regular milk), (3) CW6:4, (4) CW5:5, and (5) nitrogen-free (lactose). The milk concentration was converted from a human equivalent dose (400 mL/60 kg body weight/day). All the milk-administered groups showed significantly greater growth performance, including body weight and weight gain compared to the isocaloric lactose control (*p* < 0.05). However, different CW ratios in milk had no effect on growth performance. Additionally, body composition, i.e., lean body mass and adiposity, was not affected by the CW ratio. Interestingly, CW6:4 and CW5:5 had significantly higher plasma branched-chain amino acids concentrations than control and CW8:2 (*p* < 0.05). In addition, CW5:5 showed significantly increased grip strength by 12–24% and time to exhaustion by 8–62% compared to the other groups (*p* < 0.05), indicating that the higher whey proteins ratio improved physical performance. We concluded that whey proteins-fortified milk enhances muscle strength and endurance exercise capacity without altering lean mass in rats.

## 1. Introduction

Milk plays an important role in human nutrition as a source of various essential nutrients such as protein and minerals [1]. More than six billion people worldwide consume milk and dairy products [2]. Milk consumption provides a wide range of nutritional values to children, adults, and the elderly. Milk proteins, in particular, are considered functional foods and have been reported to affect growth, bone health, weight control, and even chronic diseases [3]. As consumers are increasingly aware of the importance of protein consumption for health, milk has been emerging as a convenient protein source.

Cow’s milk has a unique protein composition in that casein and whey proteins account for 80% and 20% of milk protein, respectively [4]. Both casein and whey are nutritionally high-quality proteins compared to plant-based proteins due to their complete essential amino acid profiles and high digestibility [5]. Casein is the majority of milk protein and consists of αs1, αs2, β, and κ-casein [6]. Casein, a so-called slow protein, is slowly digested since it is coagulated under low pH conditions [7]. Many previous studies reported that casein increased calcium absorption and bone mineral density [8,9]. Moreover, casein inhibits muscle protein breakdown and contributes to moderate but prolonged muscle protein synthesis [10]. Whey proteins are mainly composed of β-lactoglobulin and α-lactalbumin with bovine serum albumin, immunoglobulins, and lactoferrin [6]. Whey proteins are soluble protein fractions from milk that rapidly pass through the stomach and release amino acids into the blood [7,11]. The functional properties and health-promoting aspects of whey proteins have been reported. For instance, whey proteins exhibit excellent functional properties such as gelation, emulsification, and foaming [12]. Additionally, whey proteins exert health-promoting effects, including antioxidant, anti-inflammatory, and antiviral activities [13]. Moreover, whey proteins play an important role in increasing lean mass and enhancing exercise endurance. Previous studies reported that whey proteins are superior to stimulating muscle protein synthesis due to their higher leucine content than other proteins [10,14]. Furthermore, whey proteins resulted in higher maximal muscle strength and lower muscle fatigue than casein [15,16].

Having integrated the above information, it appears that blending casein and whey proteins may create a synergistic benefit by complementing each other. Previous studies investigated the effects of the addition of casein or whey proteins for the physicochemical properties of food [17,18,19]; however, few studies have investigated the effect of adjusting the ratio of casein to whey proteins (CW) in milk on the protein quality and biological activities [20,21,22]. Our previous study demonstrated that CW5:5, the adjusted protein composition in the diet, improved protein quality in rats compared to other ratios [20]. Additionally, CW4:6 reduced the allergenicity compared to CW8:2, which is the conventional ratio of cow’s milk [21]. Moreover, CW ratios in goat milk affected appetite by modulating brainstem and hypothalamic neuronal activation in mice [22]. Nonetheless, to the best of our knowledge, no studies have been conducted on the effects of CW ratio in milk on muscle mass, strength, and exercise performance. Therefore, we evaluated whether adjustment of CW ratio in milk alters body composition, muscle strength, and endurance exercise capacity in rats. We also compared the effects of milk supplementation with non-milk supplemented control (isocaloric lactose supplemented).

## 2. Materials and Methods

### 2.1. Sample Preparation

Milk samples were manufactured by Maeil Dairy Industry Co., Ltd. in Seoul, Korea. Briefly, fresh skim milk was microfiltered to fractionate milk using equipment (TetraPak, Silkeborg, Denmark) with 0.1 μm polyvinylidene fluoride spiral wound filters (Synder Filtration, Vacaville, CA, USA) and processing pressures (concentration factor 4, inlet pressure 50 kPa, and outlet pressure 100 kPa). The retentate and permeate were mixed, considering the respective casein and whey proteins content, to obtain milk samples with adjusted CW ratios. The samples were ultrafiltrated using a 0.05 μm spiral membrane (Alfa Laval, Lund, Sweden). All samples were prepared with a 2% fat content by adding cream produced when making skim milk. Then, samples were sterilized for 2 s at 130 °C with 15,000 kPa, freeze-dried, and stored at −80 °C. The proximate analysis of the milk sample was carried out using AOAC methods such as oven-drying for moisture analysis, muffle furnace for crude ash, micro-Kjeldahl for crude protein, and Soxhlet extraction for crude fat [23]. Carbohydrates (%) were determined by subtracting the moisture, crude ash, crude protein, and crude fat from 100%. Energy values were obtained using the content of protein, fat, and carbohydrates supplying 4.27, 8.79, and 3.87 kcal/g, respectively [24]. The composition of milk with adjusted protein ratios is shown in Table 1.

### 2.2. Animals and Experimental Design

This study was approved by the animal ethics committee of Chemon Inc. (Yongin, Korea) (20-RE-0687N). Four-week-old male Sprague-Dawley rats (with average body weight 104.1 g) were obtained from Orientbio Co. Ltd. (Seongnam, Korea). They were housed in controlled conditions (23 ± 3 °C, relative humidity at 55% ± 15%, and 12 h light/dark cycle); feed (AIN-93G, Saeronbio, Uiwang, Korea) and water were available ad libitum. After three days of acclimation, the rats were randomly assigned to five groups with six in each group (*n* = 6): (1) control, (2) CW8:2, (3) CW6:4, (4) CW5:5, and (5) nitrogen-free (N-free). Lyophilized milk of various compositions (CW8:2, CW6:4, and CW5:5) was dissolved in saline and provided to rats by oral gavage (10 mL/kg body weight) for eight weeks in the same dosage as the recommended daily intake for humans (2 cups = 400 mL), given in the previous report [25]. The control and N-free groups were administered lactose (4 kcal/g) (Lynn Dairy & Lynn Proteins, Granton, WI, USA), which is isocaloric as CW8:2.

### 2.3. Growth Performance and Body Composition

Body weight and feed intake were measured once per week. The feed efficiency ratio was calculated as the ratio of body weight gain to the total feed intake (FER = body weight gain (g)/feed intake (g)) during eight weeks. At the end of the experiment, the rats were fasted overnight and anesthetized with 10 mg/kg body weight (bw) xylazine (Bayer Korea, Seoul, Korea) and 100 mg/kg bw ketamine (Yuhan Co., Seoul, Korea). Body composition was assessed using dual-energy X-ray absorptiometry (DXA) on the day of sacrifice (InAlyzer, Medikors, Seongnam, Korea). The whole body of the rat was measured by DXA at low and high energy, and the images were separated into tissues and bones. The radiograph of the body classifies high-, medium-, and low-density fat into red, yellow, blue colors, respectively.

### 2.4. Protein Digestibility

Rats were housed individually in polycarbonate cages for five days at the fifth week to collect the feces. To prevent contamination, collected feces from each rat at the fifth week were weighed and dried at 80 °C. The fecal nitrogen content was determined by the Kjeldahl method using milled feces. True digestibility was calculated as follows:

True digestibility = (Nitrogen intake − (Fecal nitrogen − Endogenous fecal nitrogen))/Nitrogen intake × 100(1)

The endogenous fecal nitrogen content was determined by the nitrogen content in feces of the N-free group. 

### 2.5. Plasma Amino Acids Concentration

After seven weeks, the blood was taken 60 min after the milk sample was administered. Blood samples were collected into ethylene-diamine-tetra acetic acid tubes and centrifuged at 1500× *g* for 15 min at 4 °C. One hundred microliters of plasma was deproteinized using 10 mg 5-sulfosalicylic acid on ice. After centrifugation, the supernatant was collected and filtered using a 0.22 μm syringe filter. Plasma amino acids profiles were determined by high-performance liquid chromatography (HPLC). The HPLC system consisted of a Dionex Ultimate 3000 (Thermo Fisher Scientific, Waltham, MA, USA), Agilent 1260 infinity fluorescence detector (Em 450 nm, Ex 340 nm) (Agilent Technologies, Santa Clara, CA, USA), a UV detector (338 nm), and an Inno C18 column (4.6 mm × 150 mm, 5 μm) (Youngjin Biochrom, Seongnam, Korea) at 40 °C. Separation was performed with 40 mM sodium phosphate (pH 7) (solvent A) and water/acetonitrile/methanol (10: 45: 45, *v/v*) (solvent B). The program was as follows: 5% B as initial conditions; 3 min, 5% B; 24 min, 55% B; 25 min, 90% B; 31 min, 90% B; 34.5 min, 5% B; 35 min, 5% B. The flow rate was 1.5 mL/min, and the sample injection volume was 0.5 μL. The amino acids were detected according to retention time compared with the standard amino acids (Agilent PN 5061-3330, Agilent, Andover, MA, USA).

### 2.6. Grip Strength Test

At the fourth and eighth weeks of the experimental period, forelimb grip strength was measured by a grip strength meter equipped with a pull bar (Bioseb, Chaville, France) 60 min after administration. The rats were allowed to hold the grid and gently pull back until their forelimb released the bar, in which the grip strength was recorded. The procedure was repeated three times, and the highest values were recorded for each rat.

### 2.7. Weight-Loaded Swimming Test

The weight-loaded swimming test was performed to evaluate exercise endurance capacity 60 min after the eighth-week administration. Each rat was placed in a swimming pool maintained at 23 ± 2 °C. A load corresponding to 5% of each bodyweight was attached to the tail. Swim training was conducted over two days for 10 min each day. Swimming times were recorded from beginning to exhaustion, as determined by failure to return to the surface within 5 s.

### 2.8. Statistical Analysis

The data are expressed as the mean ± standard error of the mean (SEM). GraphPad Prism 9 (GraphPad Software, San Diego, CA, USA) was used to analyze the data. Grip strength was analyzed by two-way ANOVA with time and supplement as within-subject factors. Other results were evaluated by a one-way ANOVA, followed by Tukey’s post hoc test. Differences between groups were considered statistically significant at *p* < 0.05.

## 3. Results and Discussion

### 3.1. Growth Performance

**Figure 1:**
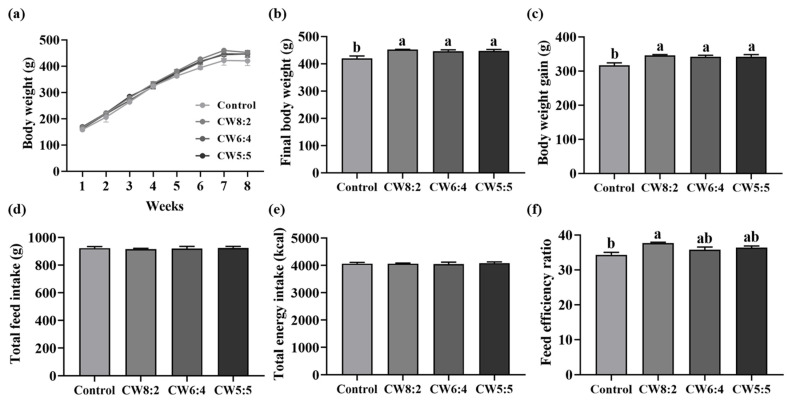
The growth performance of rats administered milk with adjusted casein to whey proteins ratio for eight weeks (*n* = 6): (**a**) weekly body weight (g), (**b**) final body weight, (**c**) body weight gain (g), (**d**) total food intake (g), (**e**) total energy intake (kcal), and (**f**) feed efficiency ratio. Data are expressed as mean ± SEM. The values with different letters indicate significant differences at *p* < 0.05. CW, casein:whey proteins.

The traits of growth performance are shown in Figure 1. The body weight and weight gain of the rats in all the milk-administered groups significantly increased compared to those in the control group without alteration of feed or energy intake. The feed efficiency ratio was significantly higher (*p* < 0.05) in the CW8:2 than in the control rats. These findings agreed with previous studies in which men and women who consumed approximately 700 mL of milk daily for twelve weeks gained 0.6 kg of body weight compared to the control [26]. Additionally, in a cohort study of children, skimmed and 1% low-fat milk intake increased weight gain [27]. This finding may be associated with the fact that milk contains high-quality protein and growth factors such as insulin-like growth factors [28]. Thus, milk consumption can help physical growth.

Nevertheless, the CW ratio did not show a significant difference in growth performance among the milk-administered groups. Similarly, in our previous study, we reported that different CW compositions of the experimental diet did not alter body weight and weight gain [20]. Additionally, Yajima et al. demonstrated that body weight gain did not change with CW ratio in rats [29]. In summary, milk administration increased body weight and weight gain; however, the different CW ratios did not show a further effect over CW8:2.

### 3.2. Body Composition

**Figure 2:**
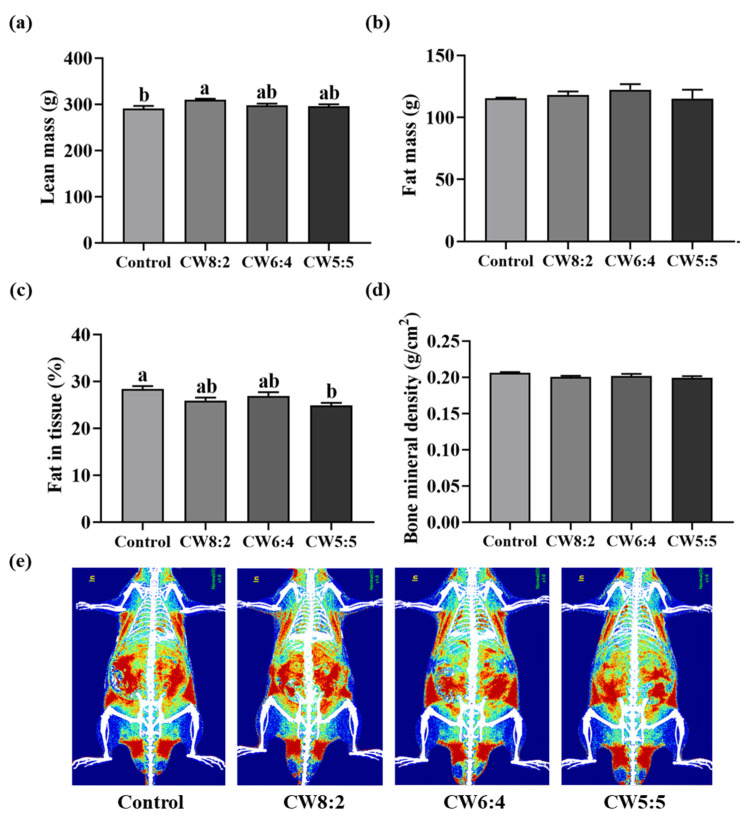
The body composition of the rats administered milk with adjusted casein to whey proteins ratio for eight weeks (*n* = 6): (**a**) lean mass (g), (**b**) fat mass (g), (**c**) fat in tissue (%), (**d**) bone mineral density (g/cm^2^), and (**e**) radiograph. Data are expressed as mean ± SEM. The values with different letters indicate significant differences at *p* < 0.05. CW, casein:whey proteins.

We performed DXA to evaluate the effect of milk with various CW ratios on body composition (Figure 2). The lean mass of the CW8:2 group was increased by 6.7% compared to the control group (*p* < 0.05). Additionally, there was no significant difference in fat mass. Fat in tissue of the CW5:5 group significantly (*p* < 0.05) decreased by 12.4% compared to that of the control. Additionally, no difference was observed in bone mineral density for all groups. 

Similarly, previous studies revealed that milk could improve body composition by increasing lean mass [30,31]. We hypothesized that muscle lean mass would increase as the whey proteins proportion increases, because it was reported that whey proteins promote muscle protein synthesis compared to casein [10,14,32]. Leucine enriched in whey proteins stimulates muscle protein synthesis as a trigger through a signaling pathway, including the protein kinase mammalian target of rapamycin [33]. However, unlike our expectations, feeding CW6:4 and CW5:5 did not significantly increase lean mass compared to the control. Several previous studies have reported that leucine concentration and muscle protein synthesis are not proportional [34,35,36]. Likewise, the difference in leucine content of the milk samples and plasma leucine concentration did not affect lean mass in this study.

There is compelling evidence that whey proteins exert anti-obesity effects in obese rats and humans [37,38,39]. Interestingly, feeding CW5:5, the highest whey proteins proportion, produced a significant reduction in fat mass in normal rats. These results suggest that feeding CW8:2 helped increase muscle mass, and CW5:5 was effective in reducing body fat compared to the control. However, the different CW ratios did not modulate the body composition in milk-administered groups.

### 3.3. Protein Digestibility

We evaluated the effect of the adjusted CW ratio on protein digestibility. The results showed no significant changes in nitrogen intake, fecal nitrogen, and true digestibility (Table 2). Previous studies showed that whey proteins are digested more easily than casein. For instance, infant formula with CW4:6 showed higher in vitro digestion than CW6:4 and CW8:2 [40]. The increased viscosity or firmness of the coagulant was observed as the ratios changed from CW0:10 to CW8:2 [41]. Our previous study’s findings also showed that CW2:8 significantly increased true digestibility compared to CW10:0, CW8:2, and CW5:5 in rats [20]. In contrast to previous studies, we observed no significant difference in the true digestibility among all groups. This discrepancy occurred because the previous study modified the protein composition of the diets, whereas we altered the protein composition in a small amount of milk. Collectively, because of the presence of casein in the basal chow diet (20% protein), the digestibility of additional milk did not affect overall protein digestibility.

### 3.4. Plasma Amino Acids Concentration

We examined each amino acid concentration in plasma (Table 3). The ingestion of milk proteins resulted in a rise in branched-chain amino acids (BCAAs) concentration, which peaked at 60 min [42]. Plasma BCAAs including isoleucine, leucine, and valine concentrations in rats fed CW6:4 and CW5:5 were significantly higher than in the control and CW8:2 (*p* < 0.05). In addition, rats fed CW6:4 and CW5:5 showed significantly elevated plasma essential amino acids and methionine concentrations compared to the control (*p* < 0.05). Milk-administered groups showed significantly higher phenylalanine concentration than the control (*p* < 0.05). However, threonine and lysine concentrations were not altered by treatments.

Postprandial amino acid profiles may reflect the amino acid composition, digestion, absorption, and metabolic rates of proteins [7]. Previous studies have shown that whey proteins contain higher BCAAs contents and are digested faster than casein [11,43]. In addition, whey proteins contain high amounts of sulfur-containing amino acids such as methionine [44]. Due to the higher BCAAs and methionine concentrations, rats fed CW6:4 and CW5:5 showed significantly higher essential amino acid concentrations compared to those in other groups. In summary, rats fed CW6:4 and CW5:5, which have a relatively high proportion of whey proteins, showed significantly higher branched-chain amino acids concentrations than control and CW8:2 rats. 

### 3.5. Grip Strength Test 

**Figure 3:**
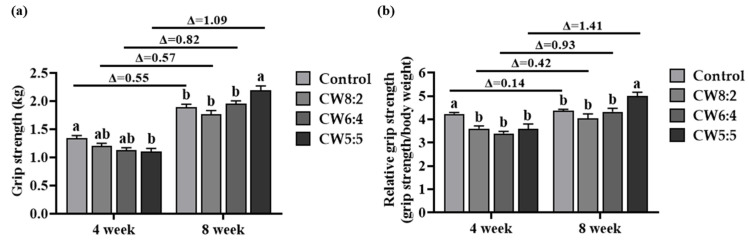
(**a**) Absolute and (**b**) relative grip strength at weeks 4 and 8 in rats administered milk with adjusted casein to whey proteins ratios for eight weeks (*n* = 6). Data are expressed as mean ± SEM. The values with different letters indicate significant differences at *p* < 0.05. CW, casein:whey proteins; ∆, difference between grip strength at weeks 4 and 8.

To investigate the effect of milk with an adjusted CW ratio on skeletal muscle strength, we measured grip strength at weeks 4 and 8 (Figure 3a). The milk administration did not change the grip strength for the first four weeks. The grip strength of the control rats was rather significantly increased compared to that of the rats fed CW5:5 (*p* < 0.05). In the final week of the experiment, the grip strength of the rats from all groups dramatically increased compared to the fourth week. Strikingly, rats fed CW5:5 showed significantly increased grip strength by 1.16-, 1.24-, and 1.12-fold compared to the control, CW8:2, and CW6:4 rats, respectively (*p* < 0.05). A two-way ANOVA analysis indicated a significant interaction between treatment and time for grip strength (time effect: *p* < 0.001, supplement effect: *p* < 0.05, time × supplement interaction effect: *p* < 0.001). The relative grip strength was calculated as the ratio of grip strength to body weight to normalize grip strength (Figure 3b). At week 4, the relative grip strength was significantly higher in the control group than in the milk-administered groups (*p* < 0.05). Rats fed CW5:5 showed significantly higher relative grip strength than control, CW8:2, and CW6:4 rats at week 8. In addition, there was a significant interaction between treatment and time for relative grip strength (time effect: *p* < 0.001, supplement effect *p* < 0.01, time × supplement interaction effect: *p* < 0.01).

These results suggest that four weeks is too short to improve muscle strength with milk supplementation. Instead, lactose was effective in enhancing muscle strength in a short period. Lactose can be used for energy or glycogen synthesis in the liver and muscle by dividing into glucose and galactose and transforming into glucose-1-phosphate [45]. Liver and muscle glycogen contents positively affect exercise performance [46,47,48]. Although all treatments contain lactose, the control group was administered a higher amount of lactose than the other groups to equal the calories in the treatment. Thus, in a short period of four weeks, lactose positively affected the grip strength of the control group.

Many previous studies have shown that collaboration with carbohydrates and protein intake can improve muscle strength [49]. However, milk consumption did not alter grip strength in a clinical study [50]. In line with the previous studies, CW8:2, the traditional ratio of cow’s milk, did not increase the absolute and relative grip strength. Nevertheless, CW5:5 significantly increased absolute and relative grip strength. There is compelling evidence that whey proteins can positively affect grip strength. The increased absolute and relative grip strength was observed in the whey proteins-supplemented group compared to the non-supplemented group [51]. Moreover, when whey proteins were supplied instead of casein in a mouse model, muscle grip strength was significantly increased without any change in muscle mass [16]. Therefore, milk with a high whey proteins ratio can increase muscle strength. 

### 3.6. Weight-Loaded Swimming Test 

**Figure 4:**
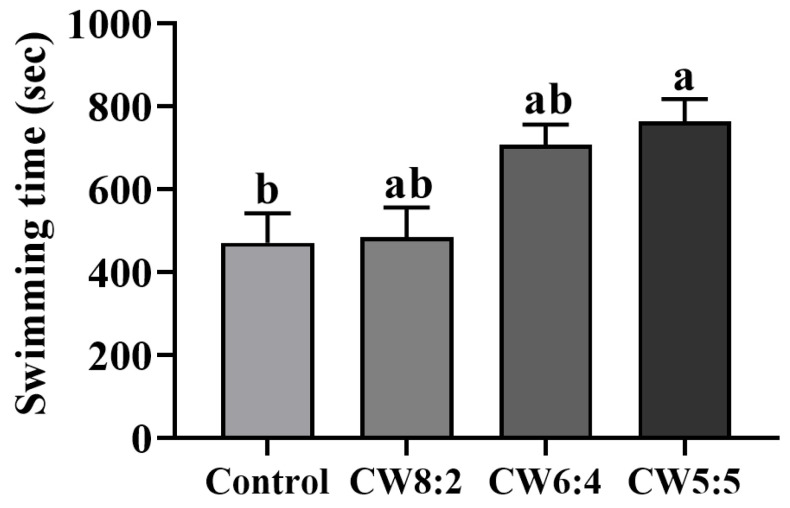
Swimming time of the rats administered milk with adjusted casein to whey proteins ratios for eight weeks (*n* = 6). Data are expressed as mean ± SEM. The values with different letters indicate significant differences at *p* < 0.05. CW, casein: whey proteins.

A weight-loaded swimming test was performed to investigate the effect of milk with adjusted CW ratio on endurance exercise capacity [52]. Swimming time for the CW5:5 group tended to be longer than for the control (*p* < 0.05) and CW8:2 (*p* = 0.051) (Figure 4). The ingestion of CW5:5 increased the time to exhaustion by 62.2%, 57.8%, and 7.9% compared to the control and ingestion of CW8:2, and CW6:4, respectively. Similarly, a previous study showed that the consumption of whey proteins increased the time to exhaustion by approximately 1.25-fold compared to the control [53]. The endurance exercise for weight-loaded swimming is mainly associated with fatigue during physical activity [54]. Fatigue during exercise is due to the depletion of muscle glycogen stores [55]. Morifuji et al. demonstrated that pre-exercise carbohydrates and whey proteins hydrolysates intake reduced muscle glycogen depletion during exercise in rats [56]. Additionally, BCAAs, abundant in whey proteins, reduced muscle glycogen depletion and delayed fatigue during physical activity in rats [57]. These results suggest that supplementation with milk with a high portion of whey proteins prolonged the swimming time to exhaustion and improved endurance capacity. 

## 4. Conclusions

In conclusion, the current findings demonstrated that whey proteins-fortified milk increased plasma BCAAs concentrations, grip strength, and endurance exercise capacity in rats. The body weight and weight gain of rats in the milk-administered groups significantly increased compared to those in the control group. However, there were no significant differences in growth performance, body composition, and protein digestibility in the milk-administered groups. Rats fed CW6:4 and CW5:5 had higher plasma BCAAs concentrations than those in the other groups. In addition, rats fed CW5:5 showed the highest grip strength and longest swimming time to exhaustion. Therefore, these results suggest that increasing the whey proteins proportion in milk above the conventional ratio (CW8:2) can enhance muscle strength and endurance exercise capacity without muscle mass accretion. Furthermore, these findings may help formulate milk for physically active people who want to maximize muscle strength and endurance exercise capacity benefits.

## Figures and Tables

**Table 1:** The proximate composition and energy values of milk powder adjusted casein to whey proteins ratio (expressed on a dry matter basis).

Item (Unit)	CW8:2	CW6:4	CW5:5
Moisture (%)	0.79	1.68	1.99
Crude ash (%)	6.45	6.26	6.15
Crude protein (%)	29.8	30.4	31.3
Crude fat (%)	17.8	16.7	16.6
Carbohydrates (%)	45.2	45.0	44.0
Calories (kcal/100 g)	459	450	450

CW, casein:whey proteins.

**Table 2:** Effect of adjusted casein and whey proteins ratio on protein digestibility in rats administered milk with adjusted casein to whey proteins ratios for eight weeks (*n* = 6).

	Control	CW8:2	CW6:4	CW5:5
Nitrogen intake (g/rat)	3.28 ± 0.16	3.53 ± 0.16	3.55 ± 0.11	3.66 ± 0.07
Fecal nitrogen (g/rat)	0.17 ± 0.01	0.19 ± 0.01	0.20 ± 0.01	0.20± 0.02
True digestibility (%)	95.5 ± 0.19	95.1 ± 0.44	94.8 ± 0.28	95.2 ± 0.42

Data are expressed as mean ± SEM. CW, casein:whey proteins.

**Table 3:** Plasma essential amino acids concentrations 60 min after administration of milk with adjusted casein to whey proteins ratios (*n* = 3).

Amino Acids (µmol/L)	Control	CW8:2	CW6:4	CW5:5
Ile	44.3 ± 0.7 ^b^	50.5 ± 2.8 ^b^	67.8 ± 0.4 ^a^	66.7 ± 2.7 ^a^
Leu	69.4 ± 2.8 ^b^	80.3 ± 3.8 ^b^	122 ± 2.7 ^a^	117 ± 2.2 ^a^
Val	90.0 ± 0.3 ^b^	105 ± 6.5 ^b^	141 ± 1.8 ^a^	132 ± 2.4 ^a^
Met	39.5 ± 1.8 ^b^	43.1 ± 0.1 ^ab^	47.2 ± 0.3 ^a^	47.4 ± 0.0 ^a^
Phe	34.9 ± 0.1 ^b^	45.6 ± 0.3 ^a^	51.0 ± 1.4 ^a^	46.9 ± 1.8 ^a^
Thr	216 ± 17	209 ± 19	202 ± 12	225 ± 21
Lys	170 ± 8.2	179 ± 17	227 ± 1.5	204 ± 17
∑BCAAs	204 ± 3.1 ^b^	236 ± 13 ^b^	331 ± 4.4 ^a^	316 ± 7.2 ^a^
∑EAAs	782 ± 8.5 ^b^	866 ± 0.8 ^ab^	1030 ± 50 ^a^	972 ± 25 ^a^

Data are expressed as mean ± SEM. The values with different letters in the same row indicate significant differences at *p* < 0.05. CW, casein:whey proteins; Ile, isoleucine; Leu, leucine; Val, valine; Met, methionine; Phe, phenylalanine; Thr, threonine; Lys, lysine; ∑BCAAs, the sum of branched-chain amino acids; ∑EAAs, the sum of essential amino acids.

## Data Availability

The data presented in this study are available within the article.
